# MR-Imaging in Osteoarthritis: Current Standard of Practice and Future Outlook

**DOI:** 10.3390/diagnostics13152586

**Published:** 2023-08-03

**Authors:** Jonathan Ehmig, Günther Engel, Joachim Lotz, Wolfgang Lehmann, Shahed Taheri, Arndt F. Schilling, Ali Seif Amir Hosseini, Babak Panahi

**Affiliations:** 1 Institute of Diagnostic and Interventional Radiology, University Medical Center Göttingen, 37075 Göttingen, Germany; jonathan.ehmig@med.uni-goettingen.de (J.E.); guenther.engel@med.uni-goettingen.de (G.E.);; 2 Clinic of Trauma, Orthopedics and Reconstructive Surgery, Georg-August-University of Göttingen, 37075 Göttingen, Germany

**Keywords:** osteoarthritis, magnetic resonance imaging (MRI), joint disease, degenerative disease, bone imaging, semiquantitative joint assessment, OA severity, OA progression, T2 mapping, Kellgren and Lawrence grading

## Abstract

Osteoarthritis (OA) is a common degenerative joint disease that affects millions of people worldwide. Magnetic resonance imaging (MRI) has emerged as a powerful tool for the evaluation and monitoring of OA due to its ability to visualize soft tissues and bone with high resolution. This review aims to provide an overview of the current state of MRI in OA, with a special focus on the knee, including protocol recommendations for clinical and research settings. Furthermore, new developments in the field of musculoskeletal MRI are highlighted in this review. These include compositional MRI techniques, such as T2 mapping and T1rho imaging, which can provide additional important information about the biochemical composition of cartilage and other joint tissues. In addition, this review discusses semiquantitative joint assessment based on MRI findings, which is a widely used method for evaluating OA severity and progression in the knee. We analyze the most common scoring methods and discuss potential benefits. Techniques to reduce acquisition times and the potential impact of deep learning in MR imaging for OA are also discussed, as these technological advances may impact clinical routine in the future.

## 1. Introduction

Osteoarthritis (OA) is the most common disease of the joint worldwide and is characterized by a multifactorial pathogenesis resulting in pain and loss of joint function. OA is considered a leading cause of disability and is associated with a high socioeconomic burden [1]_._ In 2020, 7% of the global population was affected by OA, ranking the disease 15th regarding years lived in disability worldwide [2]. Risk factors can be divided into two subgroups that interact to determine an individual’s risk for OA. Patient-specific risk factors include age, gender, obesity, genetics, and diet. Joint-specific risk factors include abnormal loading of the joint, trauma, and malalignment [3]. In addition, OA and the resulting reduced mobility can be considered a risk factor in itself, as several studies have linked it to an increased mortality from dementia and cardiovascular disease [4]. The associated economic burden resulting from treatment costs and occupational disability is estimated between 1 and 2.5% of the GNP (gross national product) in Western countries [5].

This systematic review aims to provide an overview of the value of MRI in the diagnosis of osteoarthritis. We will consider the current standard of clinical care as well as recent developments in the field. Furthermore, we will provide recommendations for structured joint assessment and analyze currently available scoring systems. Finally, we will discuss future developments in MRI and radiology itself, especially considering how artificial intelligence might reshape the landscape of MRI diagnostics.

The most frequently affected site of OA manifestation is the knee joint [6], thereby accounting for a predominant focus of scientific investigations. Consequently, this review is focused on OA of the knee, even though OA can impact various articulations in the human body [7,8]. However, most of the statements may be applicable to other joints as well.

## 2. Osteoarthritis—A Whole Joint Disease

OA has long been considered a degenerative condition primarily affecting articular cartilage. However, advances in imaging and histopathological research have led to a paradigm shift, recognizing OA as a complex joint disorder involving not only cartilage but also menisci, ligaments, synovia, subchondral bone, and periarticular muscle tissue [9].

It is widely known that ligamental tears or laxity as well as meniscal damage are associated with the development of OA [10,11]. Moreover, synovial inflammation is reported in many cases [12]. Recent studies have highlighted the critical role of subchondral bone in OA development and progression [13,14,15]. Chen et al. proposed a model describing subchondral bone loss in early OA, leading to the formation of sclerotic, less mineralized bone with altered mechanical properties and eventual disruption of the osteochondral junction in late-stage OA [13,16]. Interestingly, these changes have been observed in non-arthritic joint compartments, indicating the potential pre-eminence of subchondral bone involvement before cartilage damage occurs [13].

Furthermore, bone remodeling is significantly influenced by sex hormones [17]. In menopausal women, a study conducted by Zoli et al. revealed a notable association between osteoporosis and erosive OA [18]. Similarly, the Women’s Health Initiative conducted an extensive study, which identified a connection between self-reported OA and hysterectomy and unilateral oophorectomy. Interestingly, hormone replacement therapy in this study appeared to exhibit a protective effect [19]. Substantiating these findings, a study conducted by Jung et al. also supported the potential benefits of hormone replacement therapy [20].

Moreover, OA is frequently accompanied by impairment of periarticular muscle, which significantly contributes to functional limitations. Two major mechanisms have been identified: muscle fiber atrophy, favoring fast-twitch Type 2 fibers, and arthrogenic muscle inhibition, which refers to reduced excitability due to alterations in joint sensory receptors. It is noteworthy that atrophy in periarticular muscles is suspected to arise from chronic inflammatory processes rather than merely disuse due to pain. Histopathologic examinations found an increased amount of intramyocellular lipids probably as result of mitochondrial degeneration and fibrotic tissue between muscle fibers [9,21,22,23].

## 3. Diagnosing Osteoarthritis

Diagnosis of OA is primarily based on a thorough clinical examination of the joint, and imaging has always been important in detecting joint damage. Radiography has so far played an important role in the diagnostic process even though it is limited to the assessment of osseous structures. Additionally, patients with symptomatic OA show radiographic changes in only about half of the cases [24]. Earlier stages and potentially reversible changes of the joint can be detected by magnetic resonance imaging (MRI) which allows to assess soft tissues such as cartilage, synovia, menisci, and the surrounding muscles and ligaments [25]. However, so far MRI only plays a minor role in the primary diagnosis of OA in clinical routine, even though its sensitivity to detect structural changes in the joint has been confirmed in research settings [26].

### 3.1. Radiography

**Figure 1:**
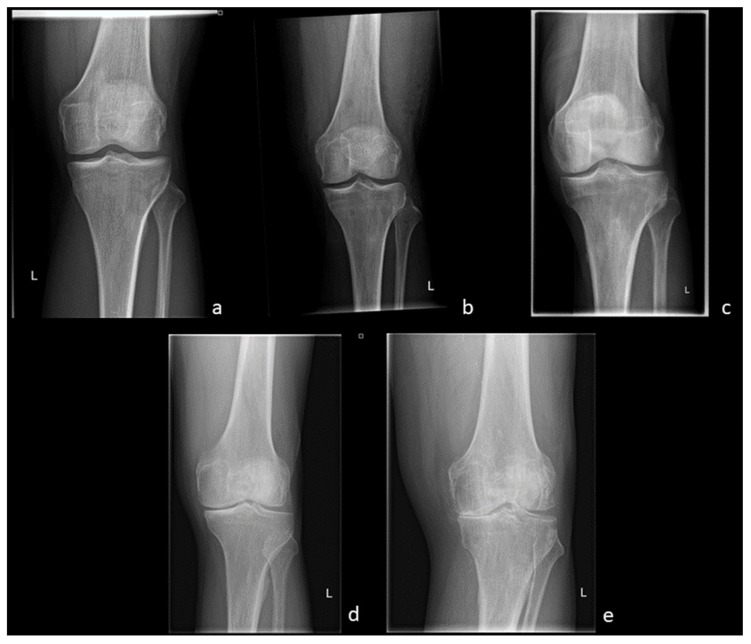
OA Stages (Kellgren and Lawrence, 1957)—(**a**) Grade 0: Physiological joint. (**b**) Grade I: Subtle JSN in the medial compartment with osteophytic lipping. (**c**) Grade II: Definite JSN in the medial compartment. (**d**) Grade III: Definite JSN in the medial compartment and sclerosis of the subchondral bone. (**e**) Grade IV: JSN with a bone-on-bone phenomenon and deformity of the medial tibial plateau as well as the medial femoral condyle.

Radiography, which is still the most commonly used imaging technique for OA, is usually acquired in two planes, i.e., the lateral and anterior-posterior view. It is widely available and inexpensive. In addition, weight-bearing images can be obtained [27]. Depending on the clinical facility and the clinical patient history, additional views, such as the patella view or the Rosenberg view, can be obtained to evaluate specific regions of the joint. Introduced by Kellgren and Lawrence in 1957, the grading of OA is still conducted on a four-grade scale (Figure 1). Grade 0 indicates the absence of OA-specific changes in the joint, Grade I is defined as doubtful OA-changes, and Grades II and III refer to minimal and moderate changes, respectively, and can be distinguished by the presence or absence of subchondral sclerosis. Finally, Grade IV considers severe stages of OA associated with joint deformity and severely reduced joint space width (JSW) [28].

OA grading on plain radiographs is based on the assessment of osseous tissues while cartilage thickness can only be evaluated indirectly as a measure of JSW [27]. However, MRI studies have shown that joint space narrowing (JSN) is not solely dependent on loss of cartilage thickness but can rather be considered as a composite of meniscal damage, meniscal extrusion, and cartilage damage [29].

### 3.2. MRI in Musculoskeletal Imaging

MRI is an established imaging technique available in most clinical institutions. Most available scanners have preset protocols. For the imaging of cartilage, 1.5 T and 3 T scanners do not differ in sensitivity for detecting pathologies. As imaging at higher field strengths results in a higher signal-to-noise ratio (SNR), spatial resolution, accuracy, and specificity are increased at 3 T [30]. Furthermore, acquisition time can be reduced at 3 T. However, it should be noted that if orthopedic hardware is implanted close to or in the imaged region, higher field strengths can lead to an increase in susceptibility artifacts caused by magnetic field inhomogeneities [31].

In addition to standard high-field MRI (HF-MRI) systems which typically operate at field strengths of 1.5 T and 3.0 T, low-field MRI (LF-MRI) systems have recently gained new attention. LF-MRI systems are available in two main configurations: standard large-bore machines and dedicated extremity scanners. Dedicated extremity scanners have demonstrated several advantages, including reduced noise and high patient comfort, making them an attractive option for focused joint examinations. In addition, these scanners have a more economical profile and offer a degree of portability, facilitating their use in different clinical settings [32,33,34].

Historically, LF-MRI has faced challenges in competing with HF-MRI regarding image resolution and contrast, limiting its diagnostic utility. However, innovative imaging protocols that exploit the unique characteristics of low-field strengths, such as shortened T1 times and longer T2 and T2* times, have significantly improved image quality. Low SNR can be addressed by applying multiple averaging which effectively increases the overall quality of LF-MRI images at the expense of longer acquisition times [33].

Despite these advances, few studies have directly compared contemporary LF-MRI systems to HF-MRI counterparts in the musculoskeletal domain. Early evidence suggests that LF-MRI performs comparably to HF-MRI in the examination of the ankle, foot, and knee. The results for shoulder imaging have been somewhat inconsistent, with certain studies reporting more management-changing results with HF-MRI. LF-MRI may be particularly suitable for acute injuries, but its sensitivity for smaller, chronic abnormalities may be limited due to lower resolution. A notable advantage of LF-MRI is its reduced susceptibility to artefacts from orthopedic hardware [33,34,35].

To ensure that patients receive accurate and high-quality diagnostic images for effective musculoskeletal management, decisions regarding the best-suited imaging modality should be made by experienced personnel. As more studies are warranted to comprehensively compare LF-MRI and HF-MRI, ongoing advancements in LF-MRI may shape the future of musculoskeletal imaging, contributing to improved outcomes. This review focuses on HF-MRI, which is more widely available due to the limitations of earlier generations of LF-MRI [32].

In addition, image quality depends on the choice of the receiver coil. Lutterbey et al. demonstrated that even in a high-field-strength scanner the image quality can be impeded by a wrong choice of coil [36]. The use of a dedicated surface coil is recommended. Furthermore, multichannel coils allow for parallel imaging, which can be used to obtain better image quality or shorter acquisition time [37].

### 3.3. MR Acquisition Protocols—The Current Standard of Clinical Care

Despite the paradigm shift towards a more holistic view of OA, the current state of imaging protocols remains tailored to evaluate the internal structures of the knee, rather than the entire joint.

The current guidelines for MRI of the knee as published by the European Society of Skeletal Radiology (ESSR) suggest the acquisition of fat-saturated proton density-weighted (PDw FS) images in three standard planes as well as a T1-weighted (T1w) image in a sagittal or coronal orientation.

**Figure 2:**
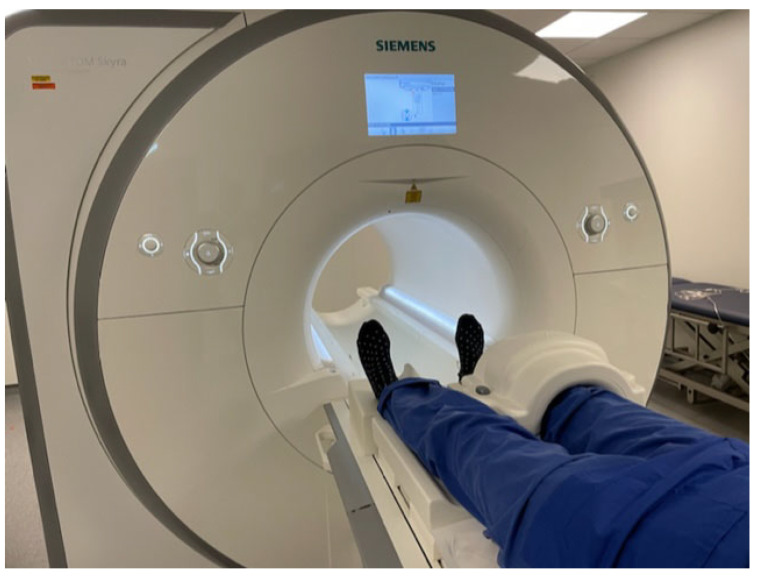
Patients enter the scanner feet first in a supine position. The coil is placed around the knee and the joints are immobilized with adequate padding.

After correct placement of the patient and the receiver coil (Figure 2), the workflow starts with the acquisition of three-plane localizers. Axial images are planned parallel to the knee joint line, while sagittal planes are planned parallel to the medial facet of the lateral condyle. Coronal planes are arranged parallel to the posterior facets of the femoral condyles. A contrast-enhanced T1w FS image may be added as an option if inflammation, such as synovitis or osteitis, or malignancy is suspected. The ESSR (Table 1) recommends a slice thickness of 3 mm for all images. The field of view (FOV) for the PDw images is recommended to be 16 cm with a matrix of 288 × 384. T1w-images should be acquired at an FOV of 18 cm with a matrix of 358 × 512 [38,39].

The acquisition of three planes is very important to ensure each compartment can be adequately evaluated. Finally, articular cartilage is best assessed in an orthogonal image. For example, while evaluation of retropatellar cartilage is limited on coronal images due to slice thickness and partial volume effects, it is easily conducted on axial or sagittal images. PDw-images allow detailed assessment of intra-articular cartilage and internal cartilage composition. However, some institutions prefer intermediate-weighted images by selecting a slightly longer echo time (TE) to benefit from the advantages of T2-weighted (T2w) sequences. These are less susceptible to magic angle artifacts and improve delineation between cartilage and synovial fluid. The overall signal on intermediate-weighted images is increased compared to T2w images [40,41]. Additional T1w images are useful for evaluation of the general anatomy, bone marrow lesions (BML), and subchondral sclerosis, as well as the search for loose bodies, that may cause locking of the joint [42]. As the T1w-images are the only non-fat-suppressed images in a standard acquisition protocol, they are particularly useful for evaluation of bone marrow and muscles that are physiologically separated by fatty tissues.

### 3.4. Fat Suppression

Fat suppression can be useful to increase contrast at the osteochondral junction. Techniques include fat saturation (FS), inversion recovery (IR) imaging, and in- and opposed-phase imaging.

FS uses a fat-specific 90° pulse immediately before the imaging sequence, tipping the protons in fatty tissues out of plane and suppressing their signal. FS relies on a homogeneous magnetic field and is very sensitive to inhomogeneities such as those caused by metallic implants. Fat suppression also has limitations owing to the signal from water in adipose tissue, and from a minor portion of fatty acids that have a resonance frequency equal to that of water. Fat saturation pulses are administered for approximately 10 ms, which can lead to a substantial increase in acquisition time. At low field strength, the difference in resonance frequencies between water and fat is reduced, resulting in incorrect tissue discrimination. Since radiofrequency pulses are tissue-specific, they can be used universally even after administration of a contrast agent [43].

IR-imaging achieves fat suppression by inverting spins along the *z*-axis and imaging at a specific time point where the protons of fatty tissues have no longitudinal magnetization at all. This technique is fast and less prone to magnetic field inhomogeneities [42]. However, the overall SNR is reduced and tissues with similar T1 compared to fat are equally suppressed.

In- and opposed-phase imaging is based on the differing precession frequencies of fat and water protons. Immediately after excitation, protons are in-phase and start dephasing until they reach a 180° dephasation. Echo time can be adapted in order to either acquire in-phase images with signal from both fat and water or opposed-phase images that show a subtraction of the two [43]. The Dixon technique is based on acquisition of both types of images and results in a pure water or pure fat image achieved by addition and subtraction of in- and opposed-phase images. Although sensitive to small amounts of fat, this technique does not suppress the signal from pure fat and is sensitive to magnetic field inhomogeneites [44,45].

Most commercially available MRI scanners today have field strengths of either 1.5 or 3.0 T, placing them in the high-field range. This allows sufficient accuracy of fat-specific radiofrequency pulses and makes FS the most commonly used technique for MRI of the joint with preserved efficacy on contrast-enhanced scans. Given its sensitivity to magnetic field inhomogeneities, IR should be considered as an alternative in the presence of metallic implants. Moreover, IR can be chosen as an effective fat-suppression technique in low-field systems. The Dixon technique can be used for the detection of small amounts of fat as well as for fat quantification, which may be helpful in case of tumors or neuromuscular disorders. In this case, as explained above, two images must be acquired, which doubles the acquisition time.

## 4. Magnetic Resonance Imaging—Common Findings

**Figure 3:**
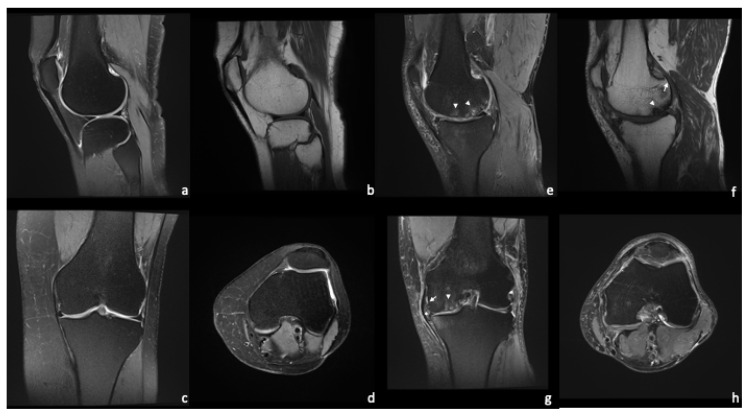
Standard protocol for knee evaluation. Healthy knee joint (**a**–**d**) vs. severe OA of the medial compartment (**e**–**h**) with BML (arrowheads), meniscal extrusion (star), and small osteophytes (arrows). The articular cartilage shows irregular thickness with some full thickness lesions (**g**).

While radiography visualizes relatively late and mostly irreversible stages of OA, MRI has the potential to detect pre-osseous deformities. A common MRI finding in OA is articular cartilage damage. Bone marrow lesions (BML) in the subchondral bone and subarticular cysts reflect extensive remodeling of the subchondral bone [13,46]. BML appear as PDw-hyperintense lesions and can be found in early OA. A study by Muratovic et al. found that BML that also appear on T1w-images are associated with late OA [47]. Subarticular bone loss and marginal osteophytes may also be seen. In the surrounding tissues, damage to the cruciate and collateral ligaments as well as the menisci may be found. Periarticular cysts, synovitis, joint effusion, and loose intraarticular bodies are considered diagnostic [12,48]. Figure 3 shows a standard MRI protocol for the knee comparing a physiological joint to a severe OA of the medial compartment.

OA has been shown to progress in different patterns, which were characterized by Deveza et al. as clinical and structural OA-phenotypes [49]. Two structural phenotypes were identified based on the extent of osteophyte formation: the hyper- and the atrophic type. The inflammatory phenotype is characterized by joint effusion and synovitis, and the subchondral type is defined by BML. The meniscal phenotype shows meniscal damage and extrusion on MRI [50]. Future treatment options may need to take these phenotypes into account and become more individualized. Therefore, careful patient selection may be required for designing of clinical trials.

## 5. Additional MRI-Techniques

### 5.1. Three-Dimensional Image Acquisition

The acquisition of 3D data sets with isotropic voxels enables the radiologist to perform multiplanar reformation (MPR) of the images, allowing the reconstruction of any desired angulation. Volumetric analysis may be performed in order to assess cartilage defects for individualized orthopedic treatment [51]. Sequences used for this purpose include Double-Echo-Steady-State (DESS), SPoiled GRadient Echo (SPGR), and Sampling Perfection with Application optimized Contrasts using different flip angle Evolutions (SPACE)-sequences.

SPGR is capable of acquiring high-resolution 3D images with nearly isotropic voxels. The technique is based on a “spoiler” pulse that dephases protons to reduce transverse magnetization and create the impression of T1w and PDw images. The disadvantage of this technique is the long acquisition time of about 10–12 min due to a low signal-to-noise ratio (SNR). In addition, being a gradient-echo sequence, it is sensitive to susceptibility artifacts [42,52].

**Figure 4:**
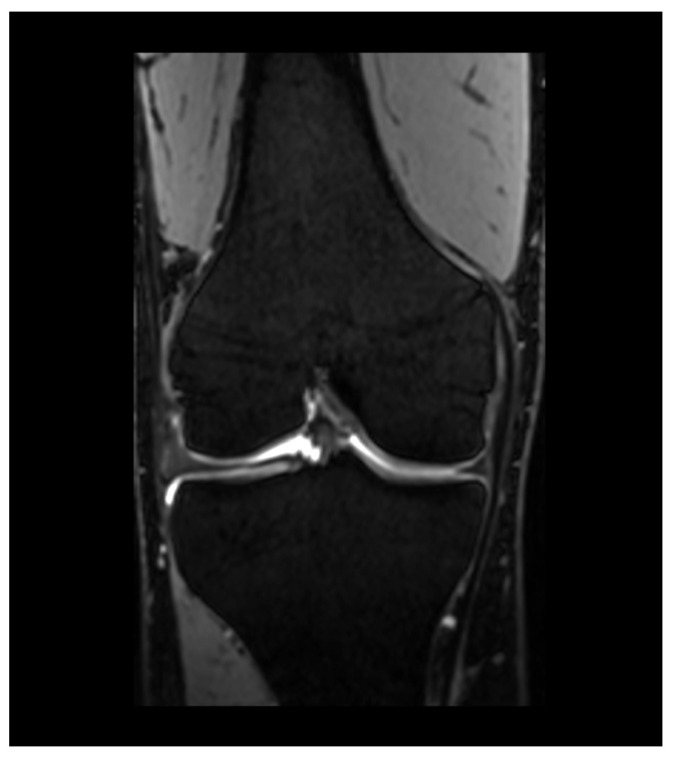
Coronal DESS image of the right knee.

DESS acquires two signals after reaching a steady state between longitudinal and transversal magnetization (Figure 4). The first is a post excitation signal after a free induction decay, while the second is acquired after a refocusing pulse. Both signals are then combined by a sum of squares. Thus, the contrast in DESS images is determined by the T1/T2 ratio [53]. DESS has the potential to calculate estimated T2 maps, and therefore may provide an opportunity to combine compositional and quantitative assessment in a single acquisition [54].

Three-dimensional-SPACE images have a higher SNR than other 3D techniques and deliver a high T2w contrast [55]. K-space under-sampling and novel parallel imaging applications have recently allowed reducing acquisition time to just under 10 min while maintaining diagnostic performance and image quality compared to a 2D standard protocol [56,57].

### 5.2. UTE-/ZTE-Imaging

A common challenge in joint imaging is the assessment of tissues with very short T2 times. Especially in OA, the assessment of short T2 tissues, such as menisci, ligaments, calcified cartilage, and subchondral bone is of paramount importance. Recently, Roemer et al. demonstrated an inferior assessment of osteophytes on MRI compared to CT [58].

Subchondral bone as the anchorage for articular cartilage is suspected to play an important role in the pathogenesis of OA. The osteochondral junction consists of the subchondral bone plate and a calcified layer of cartilage. Conventional imaging typically uses echo times in the millisecond range, making it impossible to discriminate these structures. Ultra-short-echo time (UTE) imaging and zero-echo time (ZTE) imaging are techniques used to acquire signal from these tissues, that has mostly decayed on conventional MRI. On inversion recovery UTE/ZTE-images, the osteochondral junction can be seen as a band of high signal intensity, that may appear disrupted in OA. As bone has one of the lowest T2 values in the human body, ZTE imaging can be used to accurately depict osseous tissues [59]. The images have a CT-like appearance and might have the potential to further replace bone CT with comparable resolution of 0.8–1.2 mm [60]. Furthermore, UTE imaging allows for T2*, T1, and T1rho mapping and can hence be used to evaluate cartilage and monitor therapy efficacy [61,62].

## 6. Functional Assessment on Real-Time MRI

Real-time MRI sequences allow the visualization of joint movement and function. With this technology, joint motion can be observed in real-time, providing valuable insights into joint disorders and abnormalities [63]. Research into automated bone-tracking could provide biomechanical data and potentially replace fluoroscopic or ultrasonographic examinations [64].

### 6.1. Quantitative MRI

Quantitative assessment of joint structures depends on the acquisition of 3D data sets. These techniques allow volumetric analysis of articular cartilage and measurements of cartilage thickness as well as the area of denuded bone among many others. A novel approach investigated by Bowes et al. is the calculation of a so-called “b-score”, i.e., a single parameter reflecting the change in bone shape using statistical shape modeling [65]. Cartilage volumetrics requires cartilage segmentation. This can be achieved in a time-consuming manual process. However, implementation of DL-based segmentation algorithms might facilitate this significantly in the future. Novel algorithms are based on Convolutional Neural Networks (CNN) that have been tested on both SPGR and DESS data sets [66]. Recent studies by Desai et al. showed that the four best performing CNNs were equally good at segmenting cartilage and menisci compared to manual segmentation [67].

### 6.2. Compositional MRI

The imaging techniques mentioned above focus mainly on a morphologic assessment of cartilage acquiring parameters such as cartilage thickness, volume, etc. However, several techniques have been developed in recent years to detect changes in the cartilage matrix that occur before changes in cartilage thickness and volume are detectable. Available techniques include T2 mapping, T1rho mapping, dGEMRIC, DWI (diffusion-weighted imaging), Sodium imaging, and gagCEST. Both sodium imaging and gagCEST benefit from ultra-high field strength scanners (>3 Tesla) and are hence in our opinion less likely to find their way into clinical routine in the near future.

### 6.3. T2 Mapping

**Figure 5:**
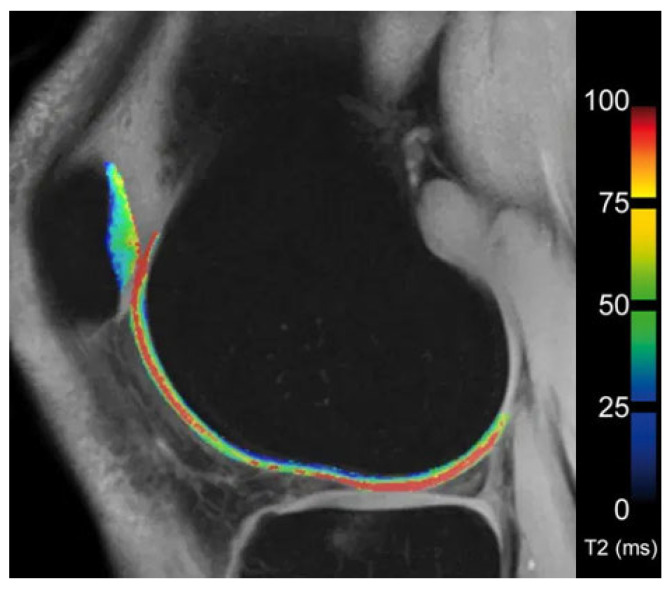
T2 map of femoral and patellar cartilage in the knee (Image kindly provided by Siemens Healthineers). The colorbar on the right indicates the T2 relaxation time.

The articular cartilage matrix comprises a collagen network and proteoglycans (PG). Early stages of OA have been shown to exhibit disruption of that matrix with a concomitant decrease in water and proteoglycans in cartilage [68]. This decrease in water content can be noticed as alteration in T2-weighted images, albeit with a lack of sensitivity [69]. Lüsse et al. demonstrated a greater sensitivity of T2-relaxation-time measurements, known as T2 mapping, for minimal changes in cartilage [70]. Several studies have confirmed the diagnostic accuracy of the technique [71]. Images are acquired via a multiecho spin echo technique to measure T2 values. Those are displayed as a grayscale or color-encoded map highlighting areas at risk for OA (Figure 5) [42,70]. T2 maps can be acquired with most commercially available scanners. However, as Koff et al. demonstrated, T2 measurements do not correlate with manifestation of OA as seen on radiography, and thus are limited for early-stage detection of OA [72].

### 6.4. T1-Rho Mapping

T1rho relaxation is also known as spin-lattice relaxation in the r(h)otational frame. In contrast to conventional T1 measurements, an additional spin-locking, long-duration radiofrequency pulse is applied, that locks spins in the transverse plane. T1rho measures the interaction between motion-restricted water molecules and their molecular environment and is sensitive to changes in the extracellular matrix [42,73]. Like in T2 mapping, color-encoded maps can be computed that display altered cartilage as an increase in T1rho relaxation time. T1rho relaxation time shows strong correlation with proteoglycan concentration but is also influenced by other factors such as collagen fiber orientation [26,74]. The disadvantages of this technique are long acquisition times and the need for special pulse sequences.

### 6.5. dGEMRIC

dGEMRIC (short for delayed Gadolinium enhanced MRI of Cartilage) is an imaging technique that requires intravenous or intraarticular administration of an anionic gadolinium (Gd)-based contrast agent. Articular proteoglycans carry a large number of negatively charged sidechains, called glycosaminoglycans (GAG). The theory is that after a fixed delay (i.e., 90 min after injection, including 10 min of joint movement), negatively charged Gd ions diffuse into the cartilage and are repelled by negatively charged GAG. Accordingly, in areas of GAG depletion, Gd ions accumulate, resulting in higher T1 values at readout. T1 mapping is one way to display the dGEMRIC indices [42]. Even though the technique is well validated, total acquisition time ranges around two hours owing to the long waiting time. Additionally, it requires contrast administration with the risk of adverse events, such as nephrogenic systemic fibrosis or anaphylactoid reaction. Some studies have also investigated dGEMRIM (delayed Gadolinium enhanced MRI of the Menisci) for the evaluation of menisci [75,76]. This technique is not well validated. A recent study by Hangaard et al. showed no correlation of meniscal damage and dGEMRIM [77].

### 6.6. DWI

These sequences first acquire a T2*w image. Then, opposing gradient magnetic fields are successively applied. This causes phase changes in the protons. Protons in healthy cartilage are relatively constrained in their motion by the surrounding matrix components. Thus, they experience two opposing gradient magnetic fields that result in a net zero phase shift. Freely moving protons such as in structurally damaged cartilage, on the other hand, acquire motion-induced phase i.e., two gradients that do not cancel each other out. DWI is to date not well validated in cartilage imaging but has the potential to monitor the efficacy of the therapy and cartilage quality. Diffusivity is expressed as the apparent diffusion coefficient (ADC). Nevertheless, ADC measurements are only relative to the previously acquired image and do not represent absolute diffusion [78,79,80,81].

### 6.7. gagCest Imaging

gagCEST, short for “glycosaminoglycan chemical exchange saturation transfer”, is a property of endogenous diamagnetic metabolites with interchangeable protons such as the negatively charged side chains of articular proteoglycans. Imaging is based on a long saturating radiofrequency pulse at the resonance frequency of interchangeable GAG protons resulting in zero net magnetization. Proton interchange occurs with adjacent water molecules until a steady state is reached. Image readout can be performed by using normal acquisition parameters, allowing for measurement of decreased water signal as a function of GAG abundance [82].

This technique has limitations due to magnetic field inhomogeneities and spillover effects, i.e., radiofrequency pulses are not highly selective resulting in an effect on water molecules. Spillover can be reduced at higher field strengths such as 7T MRI, which allows for a more selective GAG pulse. While contrast has been described as negligible at 3T, 7T enables gagCEST mapping. gagCEST may provide a useful diagnostic tool to detect onset of early OA and is sensitive to minor changes in cartilage. However, the technique has not been confirmed in larger cohort studies [83]. Identification of CEST-compatible metabolites may lead to more applications of the technique, e.g., acidoCEST-MRI that allows for determination of articular acidity [84].

### 6.8. Sodium Imaging

As previously described, PG are large molecules with negatively charged side chains. The negative charges need to be compensated by positive ones. Sodium is abundant in the extracellular space and is therefore highly concentrated in the interstitium of cartilage. Sodium—just like hydrogen—performs a precessional motion with a characteristic Larmor frequency of 11.3 MHz/Tesla and can therefore be specifically excited using radiofrequency pulses. Images can be acquired at very short TE. Limitations of the method include a low SNR owing to the lower sensitivity of radiofrequency pulses, lower abundance compared to hydrogen, and the limited hardware availability. Moreover, partial volume effects from synovial fluid can be challenging. As sodium follows a biexponential decay with very low T2 time, the technique requires acquisition at ultra-short echo times [85,86].

### 6.9. Semiquantitative Scoring Methods

In an effort to standardize joint assessment, several semiquantitative scoring methods have been established (Table 2). Going back to a time when MRI was not an option, visualization of cartilage could be achieved arthroscopically. In the 1960s, the Outerbridge classification system was introduced, which graded cartilage defects according to their depth as seen on arthroscopy [87]. With the advent of MRI, the scoring system was modified to meet the needs of radiologists. Grade 1 refers to focal areas of hyperintensity with normal cartilage contours, grade 2 includes cartilage defects up to 50% of the cartilage depth, grade 3 describes a defect >50%, and grade 4 a full thickness lesion with denudation and reactive changes of the underlying bone [88].

To create a scoring method to monitor treatment efficacy in non-OA cartilage defects, orthopedic surgeons and radiologists created the MOCART (Magnetic Resonance Observation of Cartilage Repair Tissue) score, which is currently available in its revised version, the MOCART 2.0 [89]. The score evaluates the articular surface by scoring the volume of cartilage defect filling, integration with adjacent cartilage, the surface and structure of the repair tissue, and signal-intensity of repair tissue on PD-weighted-images. The maximum score is 100 points. The intra- and interreader reliability have been demonstrated to be good for experts, albeit rather poor for inexperienced readers. The performance of inexperienced readers could be improved by using an atlas published along with the scoring method [89].

Aiming to implement a whole-organ scoring method with a focus on articular structures believed to be involved in the pathogenesis of OA, Peterfy et al. published WORMS (Whole-Organ Magnetic resonance imaging Score) in 2004. The authors defined 14 compartments of the knee and scored each compartment on a predefined scale for cartilage integrity, BML, cysts, bone attrition, and osteophytes. Additionally, synovitis/effusion, loose bodies, and synovial cysts were scored on a three-grade scale. The final score was defined as the sum of points assigned to each of these features with a maximum of 332 points [48].

A similar approach was taken by Kornaat et al. in 2005 with the introduction of the Knee Osteoarthritis Scoring System (KOSS). The KOSS is based on a simplified assessment of cartilage defects, osteophytes, subchondral cysts, BML, effusion, and meniscal lesions, but does not assess surrounding ligaments [90].

In 2008, Hunter et al. reported that the effect size and the standard response means of WORMS were small [91], resulting in the development of BLOKS (Boston–Leeds Osteoarthritis Knee Score). Here, assessment scales were adapted, which led to a better correlation with pain on the Visual Analogue Scale (VAS). BLOKS assesses nine intraarticular regions and includes eight items rated on a three-point scale [92].

Applied to data from the Osteoarthritis Initiative, BML-measurement in BLOKS turned out to be complex and somewhat redundant as well as inferior to WORMS. On the other hand, meniscal scoring was superior in BLOKS compared to WORMS [93]. Consequently, MOAKS was developed by Hunter et al. in 2011 in order to facilitate assessment and evolve existing tools towards a novel scoring method with a high interrater reliability. MOAKS considers the same 14 subregions as WORMS scored on a scale from 0–3. The scored items are BML/cysts, articular cartilage, osteophytes, Hoffa’s synovitis and synovitis-effusion, menisci, ligaments/tendons, and periarticular features [94].

All of the above scoring methods use Non-Contrast-Enhanced (NCE) Images. For synovitis, a study by Roemer et al. showed superior precision of Dynamic Contrast Enhanced (DCE)-MRI [95]. In 2011, Guermazi et al. published a semiquantitative scoring method for synovitis. The method assesses nine joint sites and grades synovitis from 0 to 2 depending on maximum synovial thickness. In the presence of loose bodies or Baker cysts, these sites are scored additionally [96].

In 2020, Roemer et al. introduced a scoring method for the rapid determination of OA phenotypes, called ROAMES (Rapid OsteoArthritis MRI Eligibility Score). The tool is based on a three-compartmental approach (PFJ—patellofemoral joint, MTFJ—medial tibiofemoral joint, LTFJ—lateral tibiofemoral joint) determining the maximum grades for cartilage lesions, BML, osteophytes, menisci, and inflammation. These items were adapted from the MOAKS and WORMS scoring systems [97].

The treatment of OA relies on the effective collaboration of several clinical disciplines. Therefore, it is important to agree on a scoring method that is understood by all involved professions. All of the above scoring methods have been shown to be highly reproducible and can be used as outcome measures in clinical trials.

**Figure 6:**
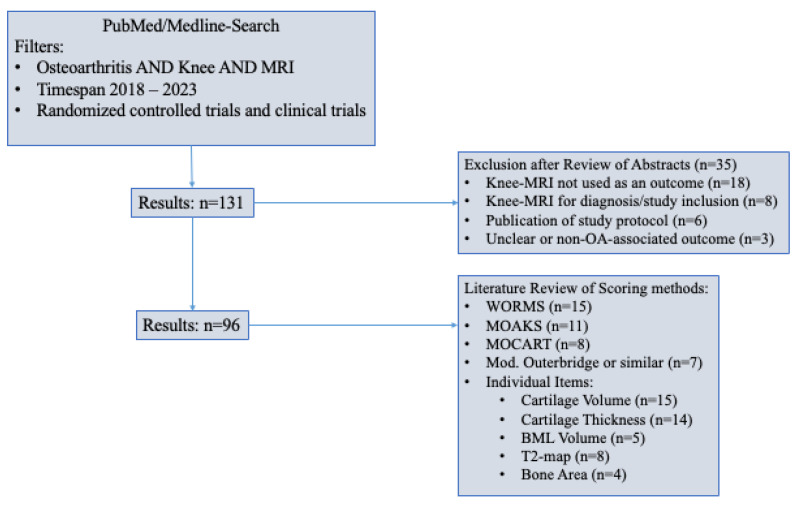
Literature review for commonly used scoring methods and MRI outcome parameters.

Here, we performed a systematic evaluation of clinical trials and randomized controlled trials over the last 5 years with MRI-parameters as an outcome measure using PubMed- and Medline-Databases. Searching for the keywords “osteoarthritis”, “MRI”, and “knee” returned 131 results, of which 97 utilized articular, Knee-MRI-based measures as an outcome parameter (Figure 6, Table S1). Publications that did not use Knee-MRI features as an outcome parameter were excluded along with studies that used MRI diagnosis as an inclusion criterion. Reports on planned, but not yet conducted trials were also excluded. Of the remaining trials, 15 relied on the WORMS and 11 on MOAKS, being the most commonly used semiquantitative scores. KOSS and BLOKS were not used in any of the trials. A four-grade cartilage assessment, like the modified Outerbridge approach, was used in seven cases. Eight studies investigating cartilage treatment used the MOCART score. Overall, most trials analyzed did not use any of the structured, semiquantitative assessments, but evaluated independent items, such as cartilage thickness (*n* = 14), cartilage volume (*n* = 15), or BML-volume (*n* = 5). Eight trials used T2 maps for measurements.

In our experience, the ICRS-approach is the most practical score for clinical reporting. However, unless otherwise established, we would recommend MOAKS for whole-organ disease monitoring, especially in a trial setting. MOCART can be used as a powerful tool to assess the efficacy of surgical treatment of chondral defects. As therapy becomes more individualized, phenotypic characterization may become more important. Currently, ROAMES can be considered as the best option for phenotyping with a sensitivity of 86% [97].

### 6.10. Reduction in Acquisition Time

Despite the different acquisition times of various protocols in use, efforts have been made to further accelerate imaging. Over the last decades, prevalence and incidence of OA showed a substantial increase due to a number of risk factors [98] creating a growing need for MRI examinations. Reducing acquisition times may lead to an increase in the total number of examinations and reduced waiting lists, which would subsequently enable further integration of MRI into clinical practice.

A first step towards faster acquisition times was proposed by Hutchinson et al. in 1988: The use of a multiple detector array coil placed around the patient to allow spatial localization of signal, and to reduce the number of phase encoding steps to encode spatial information [99]. Parallel imaging—like most acceleration techniques—is based on undersampling of k-space. The acceleration factor R represents the reduction in acquisition time. As less signal is acquired during the acquisition process with increasing R, the SNR decreases, consequently limiting the possible acceleration [100].

Simultaneous multislice (SMS) is another parallel imaging approach that allows acquisition of signal from multiple slices within one repetition time (TR). As TR is usually much longer than TE, several acquisitions can be fitted into one TR by applying multiple slice-selective excitations. Unlike the in-plane technique mentioned above, there is only a marginal loss of SNR here [101].

Further acceleration of MRI acquisition can be achieved by a technique called Compressed Sensing (CS). This relatively new approach is also based on the undersampling of k-space data. This undersampling is performed in a pseudo-random pattern acquiring more samples from the center of the k-space than the surrounding regions. This results in image noises that can be denoised via an iterative reconstruction algorithm. Luckily, CS-effectiveness is increased in 3D acquisitions, which are the most time consuming [102].

## 7. Recent Developments—The Advent of Deep Learning

Recent developments in the field of deep learning (DL) have led to another substantial decrease in acquisition time. Models can be trained with preexisting data sets enabling them to reconstruct images from undersampled data while preserving or even improving image quality [103]. Additionally, Müller-Franzes et al. found that DL has the potential to accurately quantify T2 relaxation times, resulting in an accelerated acquisition of T2 maps [104]. Other approaches use deep learning methods in order to reconstruct higher-resolution images at regular acquisition times [105]. A study by Hammernik et al. demonstrated a fourfold acceleration using this approach [106]. However, DL reconstruction has to date not been sufficiently evaluated for diagnostic accuracy, rather only for image appearance, which makes further research in the field necessary [107]. On the other hand, Johnson et al. demonstrated diagnostic equivalence of DL-reconstructed images at a twofold reduction in scan time [108].

The three techniques mentioned above can be combined to achieve further acceleration. Some vendors offer sequences that allow acquisition of a standard protocol in just below two minutes.

The increasing workload, as more scans can be performed in the clinical routine, could be compensated in the future by implementing deep learning for joint assessment into the clinical workflow. A recent publication by Kijowski et al. reviews several studies on this topic and looks forward to future developments in this field [66].

A recent study by Kulseng et al. demonstrated that CNNs are highly capable of correctly identifying joint structures [109,110]. Similar studies on cartilage lesion detection and cartilage segmentation have been very promising and with quality close to radiology fellows [111]. Although further research is necessary, another application for DL is OA risk assessment. Recent findings suggest limited diagnostic performance of existing models, but future combination of clinical and imaging information prospectively, even from different time points and modalities, is quite encouraging [66].

Despite the optimism about these new developments, future studies should focus on this topic as the existing data are still insufficient. Research is currently limited by a lack of training data sets, i.e., MRI scans that have been manually annotated to train a DL algorithm. In addition, in contrast to classic machine learning where rules are defined by a human programmer, DL algorithms define these rules themselves. These need to be carefully examined to analyze the reliability of such self-generated rulesets [111,112].

## 8. Outlook

Imaging in OA continues to evolve and will prospectively be dominated by MRI. The latest advancements in LF-MRI represent a significant stride towards enhancing MRI accessibility, particularly in middle-to-low-income countries. Detection of early changes in the joint and the surrounding tissues may allow to start curative therapies on time, halting the course of the disease and reducing the socioeconomic impact of OA. Future imaging protocols might incorporate additional sequences for comprehensive whole-organ assessments, encompassing biomechanics, cartilage composition, and surrounding tissues. Three-dimensional acquisition bears the potential to replace standard multiplane protocols that are currently in use. With the advent of deep-learning-based methods, quantitative measurements might change the way we monitor disease progression and measure treatment efficacy.

Nevertheless, deep learning may soon play an important role in image reconstruction with either increasing image quality or decreasing acquisition time substantially. Prospectively, this will lead to a greater availability of MRI and an increase in the number of examinations performed. Thus, MRI may gain further importance in the diagnostic process of OA. The increasing workload may soon be offset by deep-learning-based algorithms that can alert the radiologist to cartilage lesions or even assess the individual risk for OA progression. With deep learning currently being heavily researched and multiple approaches being explored, we can expect the landscape of MRI in OA to be reshaped soon.

## Figures and Tables

**Table 1:** Standard knee protocol as recommended by the ESSR.

Sequence	FOV	Slice Thickness	TR	TE	Matrix
Sag PD FS	160	3	3570	39	288 × 384
Cor PD FS	160	3	3570	39	288 × 384
Ax PD FS	160	3	3570	39	288 × 384
Cor/Sag T1	180	3	470	13	358 × 512
Optional CE T1 FS	180	3	470	13	358 × 512

**Table 2:** Semiquantitative scoring methods for Knee MRI.

Scoring Method	Intrarater Kappa	Interrater Kappa	Features Assessed	Number of Compartments Assessed
MOCART	0.57–0.87	0.57–1.0	volume fill of cartilage defect, integration into adjacent cartilage, surface, structure signal intensity, bony defect (overgrowth, subchondral changes)	-
WORMS		0.61–0.99 (ICC)	cartilage, BML, subarticular cysts, subarticular bone attrition, osteophytes, meniscal integrity, anterior and posterior cruciate ligament integrity, medial and lateral collateral ligament integrity, synovitis, loose bodies, and periarticular cysts/bursae	15
BLOKS		0.51–0.79	BML, cartilage, osteophytes, synovitis effusion, meniscal abnormalities, ligaments, periarticular features	9
KOSS	0.56–0.91	0.63–0.91	cartilaginous lesions, osteophytes, subchondral cysts, bone marrow edema, meniscal abnormalities, effusion, synovitis, and Baker’s cyst	9
MOAKS	0.42–1.0	0.36–1.0	BML, cartilage, synovitis, osteophytes, effusion, menisci, ligaments, periarticular features	14
ROAMES	0.92–1.0	0.85–1.0	cartilage, BML, osteophytes, menisci, inflammation (Hoffa-synivitis, effusion)	3
Synovitis score	0.67–1.0	0.67–0.92	synovial thickness	9

## Data Availability

Not applicable.

## Supplementary Materials

The following supporting information can be downloaded at: https://www.mdpi.com/article/10.3390/diagnostics13152586/s1, Table S1: Literature Review.
